# Optimizing Early Recovery Through Timely Mobilization in Nephrectomy Patients: A Case Report

**DOI:** 10.7759/cureus.56874

**Published:** 2024-03-25

**Authors:** Ghanishtha C Burile, Vaibhav Chandankhede, Yogesh Sewani, Neha Arya, Tejaswini Fating

**Affiliations:** 1 Cardiovascular and Respiratory Physiotherapy, Ravi Nair Physiotherapy College, Datta Meghe Institute of Higher Education and Research, Wardha, IND; 2 Otolaryngology - Head and Neck Surgery, Indira Gandhi Government Medical College and Hospital, Nagpur, IND; 3 General Medicine, Indira Gandhi Government Medical College and Hospital, Nagpur , IND; 4 Community Health Physiotherapy, Ravi Nair Physiotherapy College, Datta Meghe Institute of Higher Education and Research, Wardha, IND

**Keywords:** fast-track surgery, weakness in intensive care units, partial nephrectomy. laparoscopic procedure, radical nephrectomy, early mobility, prevent complications, nephrectomy

## Abstract

Nephrectomy, a surgical method involving the partial or complete removal of one or both kidneys, is performed if there is the presence of a tumor or many other reasons. In the above case, a 60-year-old female patient with a history of recurring symptoms, stomach pain, and fever, as well as a previous history of tuberculosis was brought to a tertiary care hospital. The patient underwent a left-sided nephrectomy. An X-ray and a complete blood count (CBC) were done during the investigations. Patients experienced various post-operative complications like respiratory discomfort, secretions, early fatigue, and intensive care unit-acquired weakness (ICUAW). The patient was referred for physiotherapy. Throughout the intervention, outcome assessments showed progressive improvement in lung capacity, inspiratory pressure, and quality of life scores. Goal-oriented physiotherapy was planned according to the severity of the symptoms of the patient. The physical therapy rehabilitation program in the above case was planned for six weeks focusing on symptoms like shortness of breath, early fatigue, secretions, respiratory discomfort, difficulty in maintaining good posture because of pain at the incision site, reduced mobility, and various post-operative complications. The study focuses on the efficacy of an integrated physiotherapy strategy in increasing lung compliance, secretion clearance, and overall respiratory health. Early mobilization strategies were crucial in reducing post-surgery problems, hastening functional recovery, and shortening hospital stays.

## Introduction

Nephrectomy is a surgical procedure that has been used as a treatment method for underlying medical issues like irreversible kidney damage due to chronic infection in the kidney if there is severe traumatic injury [1]. There are two methods, radical nephrectomy (RN) is defined as the removal of the whole kidney and the surrounding fatty tissue, while partial nephrectomy (PNx) is used to treat kidney cancer that has not spread to other tissue. For tiny kidney tumors, partial nephrectomy is the preferred course of treatment. RN is considered one of the primary methods for treating renal malignancies that are limited to specific organs. The prevalence of chronic renal insufficiency is higher following kidney surgery [2]. Because of the drawbacks of RN in localized renal cell carcinoma RCC, PNx is frequently more effective, and its use is growing [3,4]. When managing renal failure, including partial nephrectomies, atrophy, renal malignancy, polycystic kidney disease, and live kidney donation for transplant retroperitoneoscopy offers a number of advantages over conventional procedures. It also has a short recovery time and requires fewer analgesics and shorter hospital stays [5]. Regarding the quality of the patient's postoperative phase and the national health system, two major benefits of employing retroperitoneal robot-assisted partial nephrectomy (RAPN) are early mobilization and a brief hospital stay [6]. Robotic-assisted laparoscopic procedures have replaced open surgical methods, enabling us to accurately remove kidneys and tumors through a few tiny incisions [7]. Long-term bed rest is linked to serious sickness, which can result in decreased muscle mass, particularly in the lower limbs, increased urine nitrogen excretion (a sign of muscle catabolism), and decreased muscle protein synthesis [8].

In various articles importance of early mobilization starts when the patient is taken sitting on a chair or at the edge of the bed, followed by small movements all around the bed on the first postoperative day. Compared to typical mobilization, which consisted of walking on the first postoperative day after surgery and involved sitting in bed for three and a half hours, walking for 30 minutes, and then standing up for four hours, earlier mobilization was associated with better recovery of pulmonary function [7]. The foundation of fast-track surgery (FTS) is the idea that patients benefit from early mobilization during their postoperative recuperation [9]. Early postoperative mobilization based on a regimen of twice-daily exercises, including core stability, orthostatic training, aerobic and resistance training, and gait training, is safe and realistic and improves functional capacity [10,11]. Early mobility appears to improve functional capacity, reduce the prevalence of ICU-acquired weakness (ICU-AW), improve the number of ventilator-free days, and raise the rate of discharge to home for subjects with serious illnesses in the Intensive Care Unit (ICU) [12]. Stretching and neuromuscular electrical stimulation are two physical medicine treatments that may help mitigate some of the harmful consequences of bed rest [13]. Bed rest, along with immobilization, were appropriate postoperative care, but they are now viewed as harmful. Surgeons should recommend lifestyle changes as the first line of treatment to speed up and improve functioning [14]. If patients with serious illnesses start physiotherapy within 2-5 days of their sickness, this is called as early mobilization. Along with in-bed mobility activities, it consists of ROM exercises, sitting, standing, transfers, and gait training [15].

This case report highlights the importance of a goal-oriented physiotherapy protocol in optimizing respiratory capacity early mobility, and reducing hospital stay. By focusing on early intervention, comprehensive respiratory care, and functional rehabilitation, this protocol aims to enhance patient outcomes and improve overall quality of life. Implementation of such protocols is essential in addressing the evolving healthcare needs and optimizing resource utilization in hospital settings.

## Case presentation

Patient information

A female patient, 60 years of age, complained of pain and distress due to pus or discharge coming from her paraspinal sinuses over her left flank region. Despite the temporary relief from homeopathic treatment, she experienced recurrent symptoms alongside shortness of breath, easy fatigue, and renal tuberculosis leading to renal failure and ultimately leading to nephrectomy. Upon initiation of tuberculosis medication, her symptoms persisted, ultimately necessitating a nephrectomy. Despite the procedure, relief from symptoms was not achieved, prompting the decision for a laparoscopic left-sided nephrectomy. Post-operatively, physiotherapy was recommended to mitigate potential complications. Table 1 details the timeline of events.

**Table 1:** Timeline of events

Dates	Events
24 August 2010	Patient complaints of pus/discharge coming from paraspinal sinuses over the left flank region
15 December 2014	The patient had similar complaints and was diagnosed with tuberculosis in the kidney leading to renal failure
19 November 2019	The patient took medications for her symptoms but did not relief
3 January 2020	Laparoscopic left-sided nephrectomy was done
4 January 2020	The patient was referred for physiotherapy

Clinical findings 

The patient was intubated and received 100% inspired oxygen, along with a positive end-expiratory pressure of 5 cm of water. Additionally, bi-level positive airway pressure (BiPAP) ventilation was administered at a respiratory rate of 27 breaths per minute. Alterations in respiratory patterns, including reduced tidal volume, respiratory rate, and inspiratory flow rate, were noted. Auscultation revealed limited air intake at the upper right lung and audible crackles.

Diagnostic assessment

A complete blood count (CBC) was performed in relation to the laboratory study, and the result was a lower hemoglobin (9.6%) level, indicating anemia and PRBC (packed red blood cells) was done. Air entry was restricted during auscultation in the bilateral lower and middle zones. In the bottom zones of percussion, a dull tone can be heard. Pillows were placed beneath the heels to help reduce edema as the patient was in ICU and reduced mobility. In investigations, chest X-rays was done. A complete blood count (CBC) was performed in relation to the laboratory study, and the result was a lower hemoglobin (9.6%) level, indicating anemia. In Table 2 hemoglobin report of the patient has been presented. 

**Table 2:** Hemoglobin report of the patient

Normal haemoglobin value	Affected value of the patient
For females- 12.1 to 15.1 g/dl	9.6 g/dl (lower than normal value)

**Figure 1:**
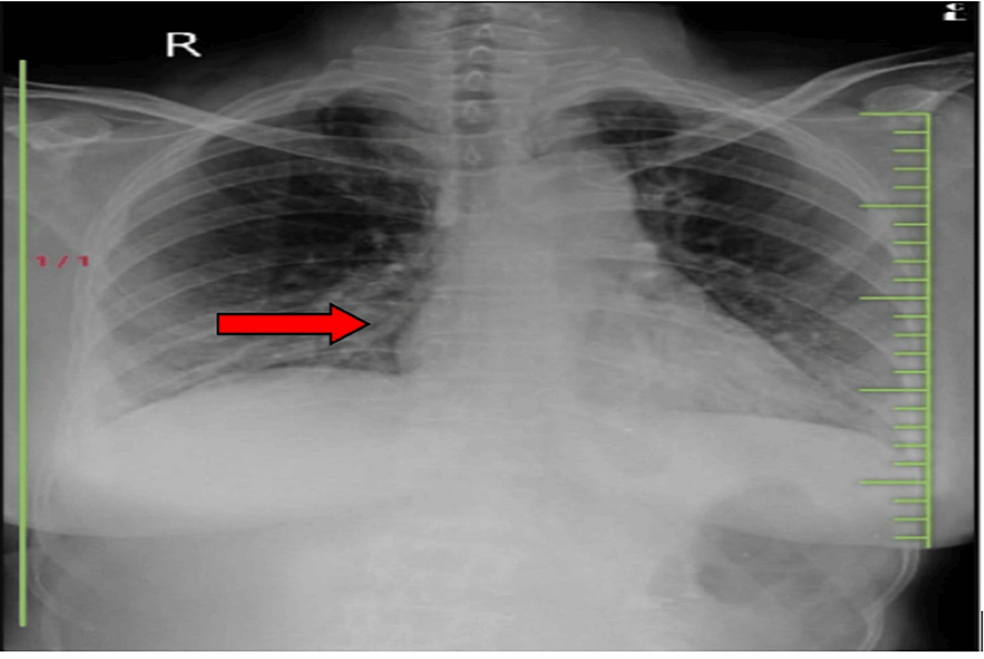
X-ray chest An X-ray revealed heterogeneous opacity on the right middle and lower zones, and prominent broncho vascular marking (red arrow).

Air entry was restricted during auscultation in the bilateral lower and middle zones. In the bottom zones of percussion, a dull tone can be heard. Pillows were placed beneath the heels to help reduce oedema. In Figure 1 an X-ray done which reveals heterogeneous opacity on the right middle and lower zones and prominent broncho vascular markings (red arrow).

Outcome measures

Patient recovery was monitored via various outcome measures, including the ICU mobility scale, lung expansion via spirometry, maximum inspiratory pressure (PImax), and quality of life (SF-36 questionnaire) as mentioned in Table 3. The progression of patient recovery was noted.

**Table 3:** Outcome measures ICU: intensive care unit; PImax: maximum inspiratory pressure

Outcome Measures	20/9/23	30/9/23	15/10/23
Lung capacity	600 cc with 1 sec holds	900 cc with 2 sec holds	1200 cc with 4 sec holds
PI max (maximum inspiratory pressure)	50 mm Hg	55 mm Hg	60 mm Hg
Quality of life SF-36 questionnaire	Score 2	Score 4	Score-5

Physiotherapy rehabilitation protocol

The primary goal of physiotherapy rehabilitation was to treat hypoxemia and reduce dyspnoea without using accessory muscles, hence boosting lung capacity. All this would be achieved with early mobility which includes bed mobility, spot marching, ambulation, and other physiotherapy approaches. A tailored physiotherapy program was planned for six weeks focusing on reducing the symptoms mentioned in Table 4. 

**Table 4:** Physiotherapy treatment goals and management ACBT: The active cycle of breathing technique.

Problem	Goal	Intervention
The patient complains of shortness of breath due to which there is difficulty in breathing	To increase breathing capacity and reduce symptoms like shortness of breath	Diaphragmatic breathing exercises help in expanding the lungs and aid in efficient oxygen exchange to improve tidal volume.
Decreased lung expansion capacity due to weakness at diaphragmatic muscles	To improve sustained maximal inspiration	Devices like incentive spirometry help in regaining the strength of diaphragmatic muscle and function it more effectively.
Decreased lung ventilation	To facilitate lung ventilation	Upright sitting has several benefits, including increased forced expiratory flow, improved oxygenation with less need for more oxygen, increased lung volume and capacity, and higher diaphragmatic excursion.
Reduced ventilation-perfusion ratio	To improve ventilation	Neurophysiological facilitation of respiration (NPF) -Peri oral stimulation.
Reduced ability to move freely and independently	To make the patient move independently	Spot marching improves balance and core stability.
Difficulty in removing cough and removal of secretions	To remove cough and secretions and clear airways	ACBT enhances lung function without increasing hypoxia or obstructing airflow -Positive expiratory pressure (PEP) therapy.
Reduced respiratory muscle strength	For preserving respiratory muscle strength	Inspiratory muscle training using threshold inspiratory muscle training (IMT).
Make the patient initiate to walk	To enhance minute ventilation and cardiac output while remaining within physiologically safe limits	Greater recovery of pulmonary function was achieved with earlier mobility, which included sitting in bed for 3.5 hours following surgery, remaining in a sitting position for 30 minutes, and then beginning to walk around in the fourth hour following surgery.
Difficulty in maintaining good posture due to pain at incision site posture	To educate the patient on ergonomic advice	Educating the patient on postural correction exercises and avoiding adapted postural patterns.
Minimize the intensity of incisional pain	To relieve pain	Cold packs can lessen the intensity of pain from an incision (for 8- 10 minutes, 2-3 times/day).
Reduced endurance	To improve your endurance levels	Cycle ergometry (Arm as well as leg ergometry) 45 min (5 min of warm-up, 35 min of moderate-intensity, and 5 min of cool-down) three times/week).

**Figure 2:**
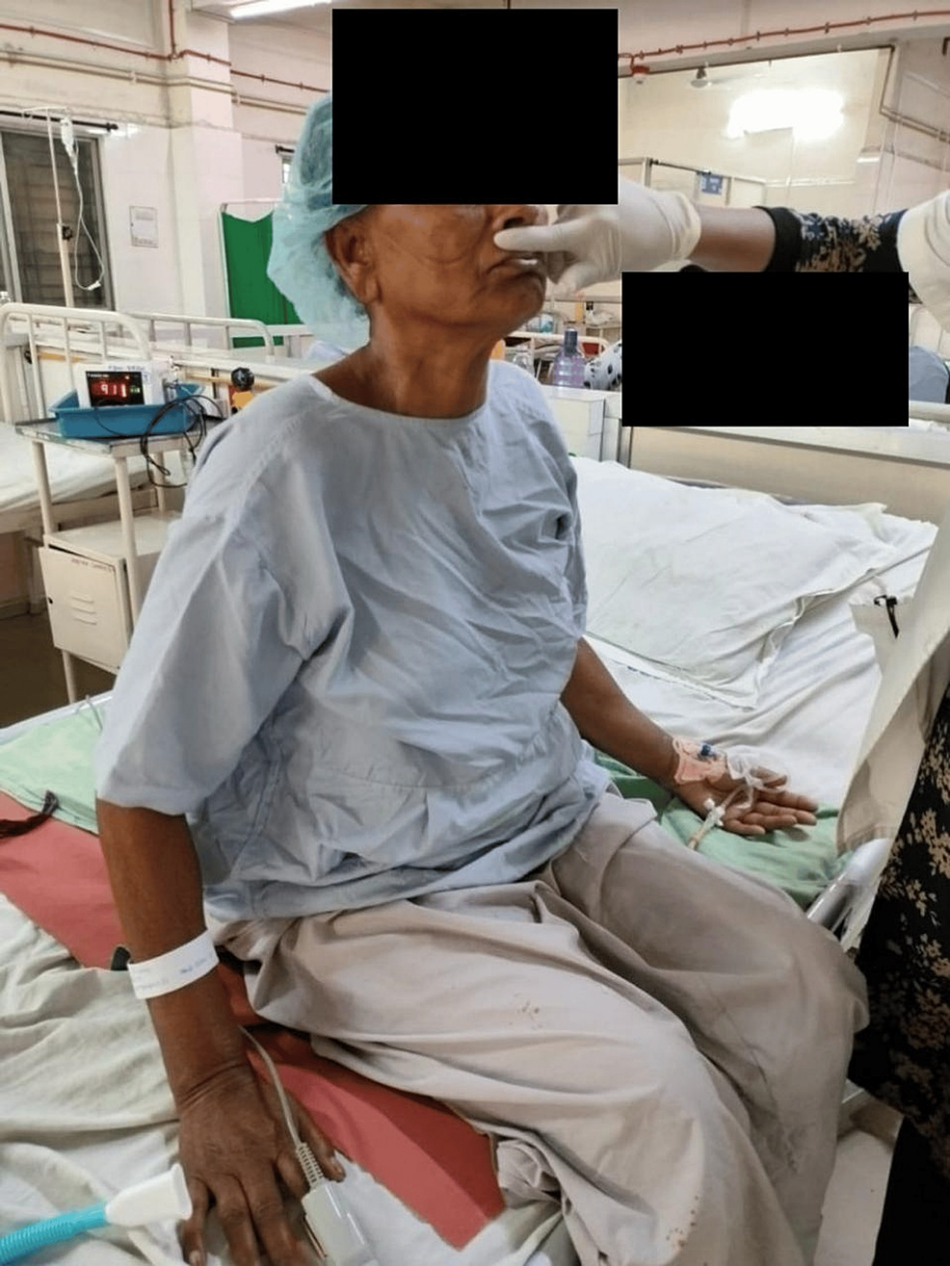
Therapist giving perioral stimulation

In Figure 2, the therapist performs neurophysiological facilitation (NPF) of a respiration technique: perioral stimulation. In this technique, the perioral pressure is delivered via the therapist's finger below the nose, on the top lip. The pressure is maintained for the duration up to which the therapist wishes the subject to breathe in an activated pattern. 

**Figure 3:**
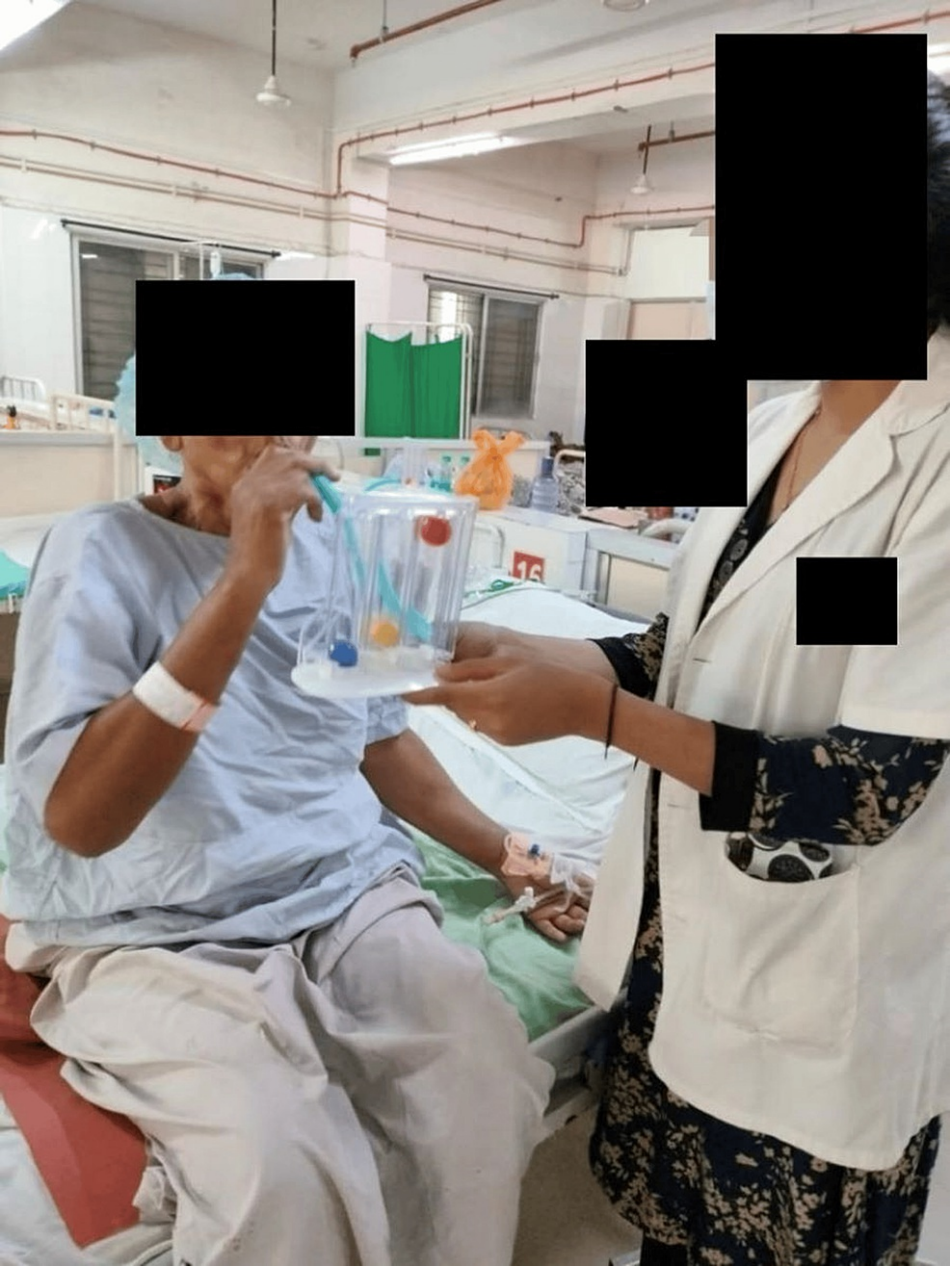
Patient performing incentive spirometry to improve inspiratory capacity

In Figure 3, the patient is performing incentive spirometry to improve inspiratory capacity to improve the strength of inspiratory muscles.

**Figure 4:**
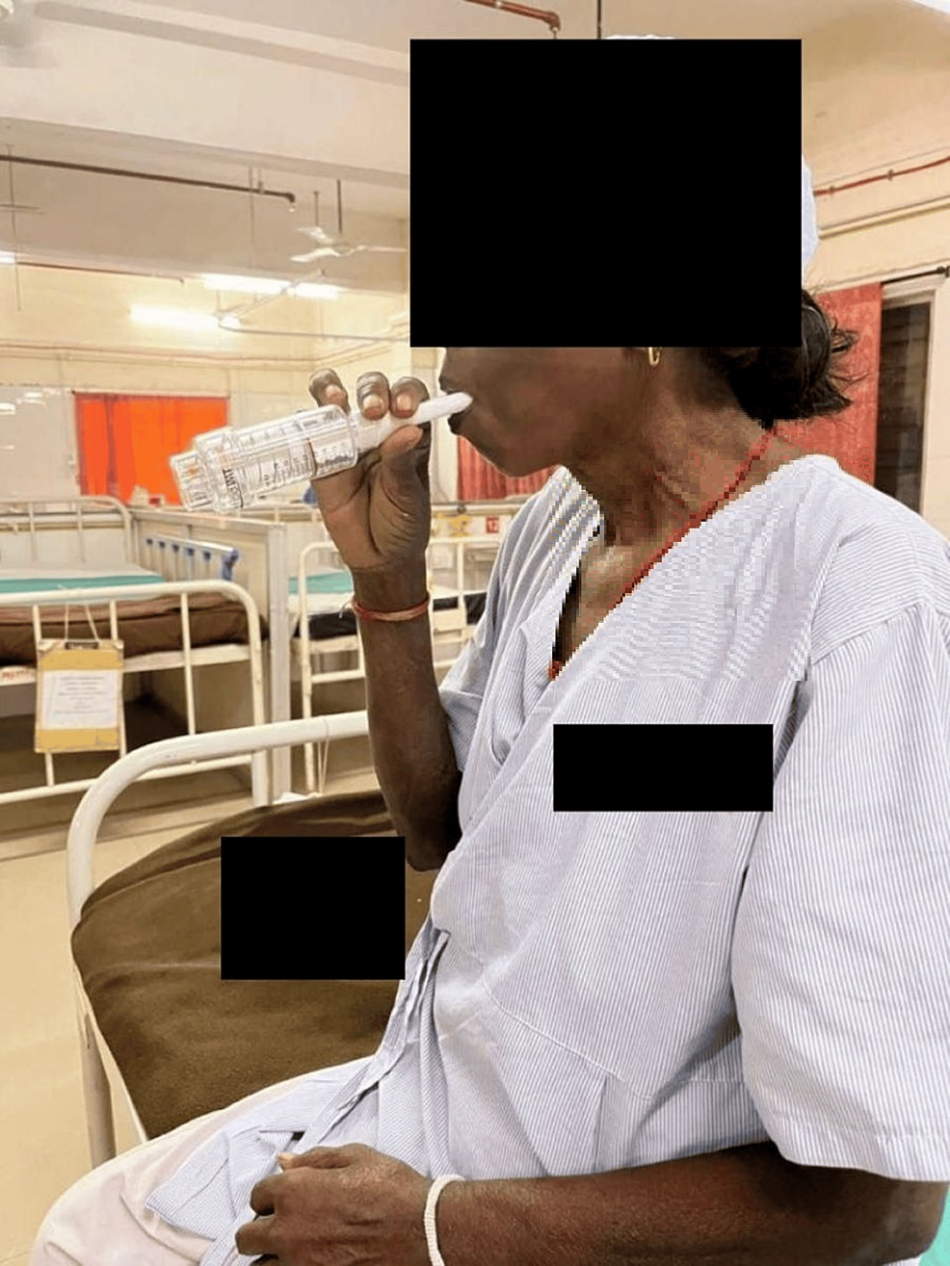
Patient using threshold IMT device IMT: Inspiratory muscle training.

In Figure 4, the patient is using a threshold IMT device to improve the strength of the inspiratory muscles and endurance of the respiratory muscles.

**Figure 5:**
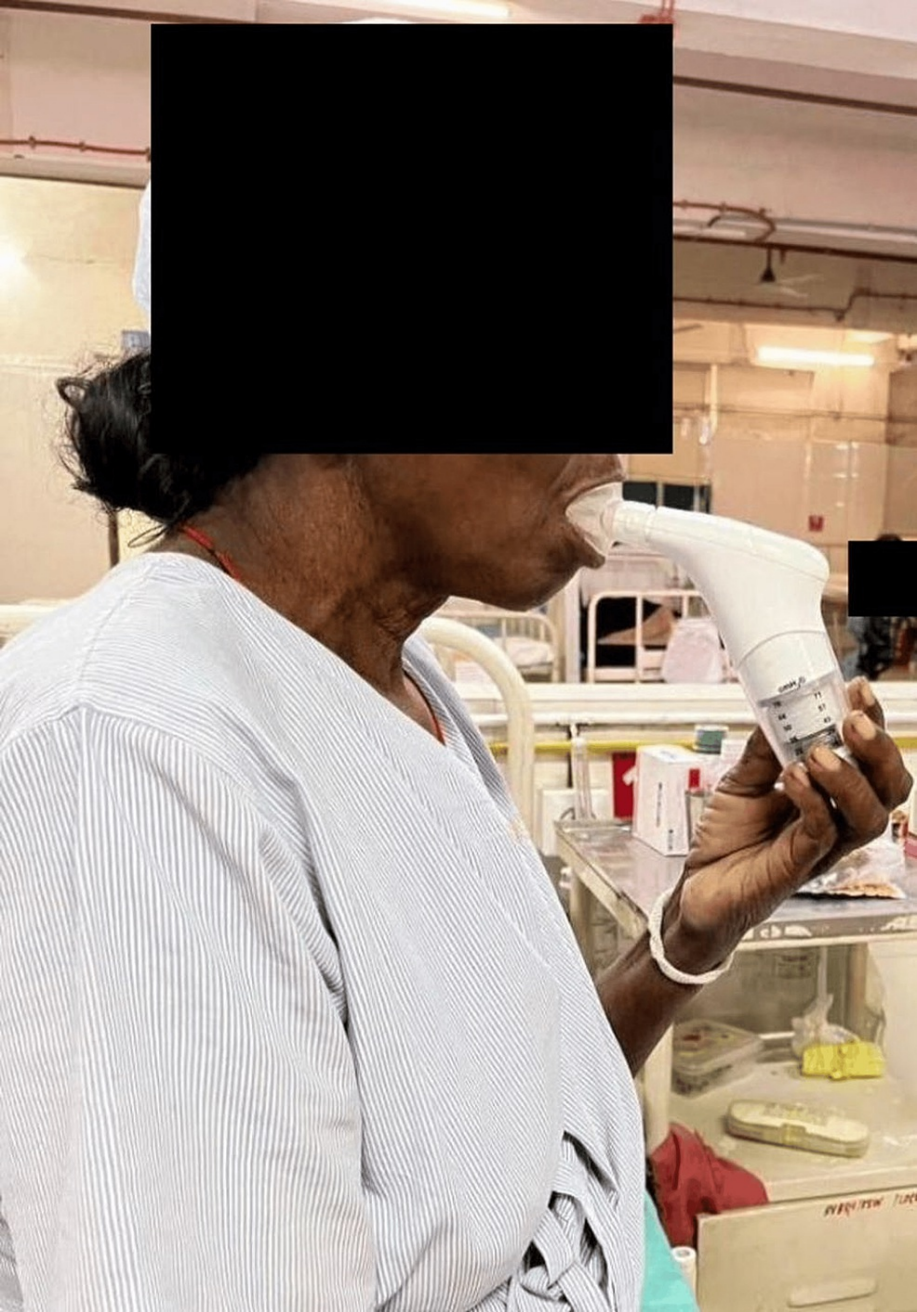
Patient using PImax device PImax: Maximal inspiratory pressure.

In Figure 5, the patient is using the PImax device to measure the improvement in respiratory muscle strength, functional capacity, and dyspnea.

## Discussion

Nephrectomy is a surgical method that can be used to treat patients with tumors in the kidney which has a profound impact on a patient's health and quality of life. In such cases, it is important to manage patients with a multidisciplinary approach. Depending on the severity of symptoms, it is imperative to devise a physical therapy protocol with early mobilization as the primary objective to ameliorate overall symptoms. The aim of physical therapy for subjects undergoing nephrectomy is secretion removal; ACBT helps decrease the viscoelasticity of secretions and ease symptoms, including dyspnoea [16,17]. The repair, regeneration, and mobility of scars after therapy have a significant impact on posture [18]. Sietsema et al. studied exercise capacity as a predictor of survival in ambulatory individuals with end-stage kidney disease suggesting that exercise training may be used to increase survival because it can change peak VO2 levels. Exercise capacity may also provide predictive information about healthy individuals with end-stage renal disease [19]. Samnani et al. examined the impact of preoperatively counseling patients on early mobilization following surgery and its role in seamless recuperation. Mobility for more than 10 minutes and from bed to a chair, on the other hand, had a significant effect [20].

In the above case, a 60-year-old female came with abdominal pain and was admitted to the hospital for evaluation. Comprehensive assessments, including CBC and X-ray, were done, leading to a recommendation for nephrectomy. Following the nephrectomy, the patient experienced various complications such as cough, shortness of breath, and easy fatigue. To alleviate these symptoms, a tailored rehabilitation program focusing on the specific complications and symptoms was designed. A series of outcome measures were used to monitor the patient's progress. These included spirometry to assess lung capacity, PI max to check for respiratory muscle strength, and the SF-36 quality of life questionnaire to evaluate overall well-being. A significant improvement was seen in reducing the symptoms of the patient. In physical therapy protocol, the primary aim was early mobilization so that the patient could perform her activities independently, other interventions that were used were breathing exercises to improve breathing patterns, reduce the symptoms of early fatigue, NPF to improve ventilation, ACBT, positive expiratory pressure (PEP) to clear airways, IMT for improving respiratory muscle strength, ergonomic advice for maintaining good posture, improve endurance via ergometry. 

Our main goal in this circumstance was to manage post-operative cardiopulmonary problems and early mobility. Physical therapy treatment aimed for early weaning from the ventilator, early mobilization, and less hospital stay. A multidisciplinary approach to improve the subject's condition was planned for improving activties of daily living and improving quality of life.

## Conclusions

The multidisciplinary physical therapy protocol was planned to reduce the patient's symptoms and increase lung compliance along with the removal of secretions, which improves air entry. A goal-directed physical intervention protocol that focuses on early mobility is depicted, which helps to decrease postoperative complications and the severity of the symptoms. Several fundamental ICU goals were accomplished during the four-week hospital stay even though complete recovery was not achievable during the treatment. Several fundamental ICU goals were accomplished throughout the patient's four weeks in the hospital despite the fact that a complete recovery was not achieved during the rehabilitation program. Early mobilization (spot marching, bed mobility training, initially ambulation with assistance) decreases the severity of the symptoms of the patient that patients may face post-operatively, improves functional recovery, and reduces hospital stays.
